# Geschlechtsspezifische Ergebnisse des Dresdner Kinder- und Jugendkopfschmerzprogrammes DreKiP

**DOI:** 10.1007/s00482-023-00756-z

**Published:** 2023-09-22

**Authors:** Laura Zaranek, Hanna Sobe, Matthias Richter, Anke Hübler, Reinhard Berner, Maja von der Hagen, Thea Koch, Rainer Sabatowski, Anna Klimova, Gudrun Goßrau

**Affiliations:** 1 Klinik für Kinder- und Jugendmedizin, Medizinische Fakultät und Universitätsklinikum „Carl Gustav Carus“, TU Dresden, Dresden, Deutschland; 2 UniversitätsSchmerzCentrum, Medizinische Fakultät und Universitätsklinikum „Carl Gustav Carus“, TU Dresden, Fetscherstr. 74, 01307 Dresden, Deutschland; 3 Klinik für Anästhesiologie und Intensivtherapie, Medizinische Fakultät und Universitätsklinikum „Carl Gustav Carus“, TU Dresden, Dresden, Deutschland; 4 Abteilung Neuropädiatrie, Medizinische Fakultät und Universitätsklinikum „Carl Gustav Carus“, TU Dresden, Dresden, Deutschland; 5 NCT Partner Site Dresden, Institut für Medizinische Informatik und Biometrie, Medizinische Fakultät „Carl Gustav Carus“, TU Dresden, Dresden, Deutschland

**Keywords:** Kinderkopfschmerz, Migräne, Edukation, Multimodale Therapie, Gruppentherapie, Pediatric headache, Migraine, Patient education, Multimodal therapy, Group therapy

## Abstract

**Hintergrund:**

Mädchen und Frauen sind häufiger von Kopfschmerzen betroffen als Jungen und Männer. Der Einfluss des Geschlechts auf die Wirksamkeit von Kopfschmerztherapien ist bisher kaum untersucht. Wir prüften geschlechterspezifische Unterschiede im ambulanten multimodalen Dresdner Kinder- und Jugendkopfschmerzprogramm DreKiP.

**Methoden:**

140 Patienten mit primären Kopfschmerzen wurden in einem 15-stündigen strukturiertem Gruppenprogramm behandelt. Zu Beginn des Programms (T0) sowie 6 (T1) und 12 Monate (T2) nach dem Ende wurden Daten zu kopfschmerzbedingter Einschränkung der Alltagsfähigkeit (PedMIDAS), Kopfschmerzfrequenz, -intensität und schmerzbedingter Alltagseinschränkung (P-PDI) erhoben. Retrospektiv wurden diese Daten für Mädchen und Jungen getrennt analysiert.

**Ergebnisse:**

Von 91 Patienten (9–19 Jahre, Median = 15; 71,4 % weiblich) lagen Daten für mindestens zwei Messzeitpunkte vor.

Mädchen zeigten zu allen Zeitpunkten eine signifikant höhere Kopfschmerzfrequenz als Jungen (Mediane Kopfschmerztage/letzte 3 Monate zu T0: ♀ 43, ♂ 20; T1: ♀ 32, ♂ 12; T2: ♀ 28, ♂ 9) sowie eine numerisch höhere kopfschmerzbedingte Alltagseinschränkung. Es zeigten sich signifikante Effekte über die Zeit mit Abnahme der Kopfschmerzfrequenz (F (2,88) = 5,862; *p* = 0,004) und Verbesserung der Alltagsfunktion (F (2,92) = 5,340; *p* = 0,006).

Eine geschlechtsspezifische Therapieantwort zeigte sich nicht.

**Diskussion:**

Therapieinhalte des DreKiP zeigten bei Mädchen und Jungen mit primären Kopfschmerzen Effekte. Höhere Kopfschmerzfrequenzen und Alltagseinschränkung bei Mädchen können vor allem hormonelle, aber auch psychosoziale Ursachen haben und sollten in Edukationsmaßnahmen aufgegriffen werden.

**Zusatzmaterial online:**

Die Online-Version dieses Beitrags (10.1007/s00482-023-00756-z) enthält die Ergebnisse der Varianzanalyse für Kopfschmerzfrequenz, Kopfschmerzintensität, PedMIDAS und PPDI im Therapieverlauf.

## Abstract (en)

**Background:**

Girls and women are more frequently affected by headache than boys and men. The influence of gender on the effectiveness of headache therapies has so far been hardly investigated. We examined gender differences in the outpatient multimodal Dresden Child and Adolescent Headache Program DreKiP.

**Methods:**

We treated 140 patients with primary headache in a 15-hour structured group program. At baseline (T0) and six (T1) and twelve months (T2) after the end of the program, data on headache-related limitation of daily activities (PedMIDAS) as well as headache frequency, intensity, and pain-related disability (P-PDI) were collected. Retrospectively, these data were analyzed separately for girls and boys.

**Results:**

For 91 patients (9–19 years, median = 15; 71.4 % female) data were available for at least two measurement time points.

Girls showed significantly higher headache frequency than boys at all time points (median headache days/last three months at T0: ♀ 43, ♂ 20; T1: ♀ 32, ♂ 12; T2: ♀ 28, ♂ 9) as well as numerically higher headache-related limitation of daily life. There were significant effects over time with a decrease in headache frequency (F (2.88) = 5.862; *p* = 0.004) and improvement in daily functioning (F (2.92) = 5.340; *p* = 0.006).

There was no gender-specific treatment response.

**Discussion:**

The DreKiP therapy shows effects in girls and boys with primary headache. Higher headache frequencies and everyday life restrictions in girls may have hormonal but also psychosocial causes and should be addressed in educational measures.

## Hintergrund

Kopfschmerzen sind die häufigste Schmerzerkrankung bei Kindern und Jugendlichen und führen häufig zu psychosozialen Einschränkungen [1]. Etwa 5 % der Kinder und Jugendlichen mit Kopfschmerzen weisen schwere kopfschmerzbedingte Einschränkungen im Schulalltag mit häufigem Schulausfall, Verlust von Bildungsmöglichkeiten bis hin zu Schulabbruch auf [2, 3]. Das trifft insbesondere für Patienten mit körperlich einschränkenden Migräneattacken zu. Bekannt ist auch, dass Jugendliche mit chronischer Migräne Aufmerksamkeitsstörungen in Abhängigkeit von der empfundenen Schmerzintensität der Kopfschmerzen zeigen [4].

In Deutschland stehen weiterhin nur wenige evidenzbasierte Behandlungsmöglichkeiten für Kinder und Jugendliche mit Kopfschmerzen zur Verfügung. Darüber hinaus gibt es auch bisher keine hinreichenden Versorgungsstrukturen für diese Patientengruppe. Es ist bekannt, dass Jugendliche mit Migräne im jungen Erwachsenenalter häufiger affektive und Verhaltensstörungen sowie Schmerzerkrankungen entwickeln [5]. Die Migräne persistiert bei der Mehrheit der jugendlichen Patienten bis ins Erwachsenenalter [6–9]. Junge Kopfschmerzpatienten weisen eine hohe Komorbidität mit weiteren chronischen Schmerzen (abdominell, Bewegungsapparat) auf [5, 10, 11]. Lebensstilfaktoren wie Koffeinkonsum, Bewegungsmangel und psychosoziale Risikokonstellationen korrelieren mit häufigen Kopfschmerzen bei jungen Patienten [12, 13].

Die gesellschaftliche Bagatellisierung von Kopfschmerzen und Migräne erfolgt in allen Altersgruppen. Das liegt einerseits an fehlendem Wissen zur Erkrankung, andererseits aber auch an bisher begrenzten wissenschaftlichen Möglichkeiten bei Priorisierung anderer Erkrankungsgruppen in der Forschungspolitik [14].

In den letzten Jahren konnten vor allem für die Pathophysiologie der Migräne wesentliche Erkenntnisse gewonnen werden. Dabei konnte zum einen die Rolle trigeminaler Nozizeptoren des peripheren Nervensystems und hier besonders des pronozizeptiven Neurotransmitters CGRP (Calcitonin Gene-related Peptide) herausgestellt werden [15, 16]. Aber auch zentralnervöse Prozesse, die eine Aktivierung des Hypothalamus als wichtigen Prozess der Migränekaskade darstellen, wurden entschlüsselt [17]. Diese Erkenntnisse zu funktionellen Korrelaten der Migräne im Gehirn und CGRP als peripher messbarem Biomarker der Migräne können zur Änderung der öffentlichen Wahrnehmung beitragen. Ziel wäre eine konsequente Diagnosestellung und Therapie für Millionen Patienten mit Migräne in Deutschland [18]. Insbesondere Mädchen mit einschränkenden Kopfschmerzen, begleitenden depressiven Symptomen und sozioökonomischer Beeinträchtigung sind nach bisheriger Datenlage besonders gefährdet für eine Chronifizierung der Kopfschmerzen und damit für eine andauernde Leistungseinschränkung [19, 20].

Die Therapie von einschränkenden Kopfschmerzen im Kindes- und Jugendalter sollte immer das individuelle biopsychosoziale Bedingungsgefüge beachten und muss deshalb interdisziplinär und multimodal erfolgen. Ziele der Therapie sind Edukation zum Kopfschmerz, Verhaltensänderungen, Verbesserung der Entspannungsfähigkeit, der Stressbewältigung und der körperlichen Aktivität. Unimodale Therapiestrategien tragen der komplexen Situation nicht Rechnung und sind damit meist nicht erfolgreich [1, 21].

Daher haben wir ein interdisziplinäres multimodales ambulantes Therapieprogramm entwickelt, bestehend aus einer Kopfschmerzschulung und einer Vielzahl von praktischen Therapien, um individuelle Ansätze für junge Kopfschmerzpatienten zu ermöglichen [22, 23]. Primäres Ziel der Studie ist es, geschlechtsabhängig therapiebedingte Veränderungen in der Kopfschmerzfrequenz und der kopfschmerzbedingten Beeinträchtigung 6 und 12 Monate nach der therapeutischen Intervention zu erfassen.

## Methoden

Therapieeffekte einer interdisziplinären multimodalen ambulanten Gruppentherapie wurden prospektiv an Kindern und Jugendlichen mit primären Kopfschmerzen untersucht. Das Studienprotokoll wurde von der Ethikkommission der Medizinischen Fakultät der TU Dresden genehmigt (Protokollnummer EK-462122017). Alle Teilnehmer und deren Eltern wurden ausführlich über die Studieninhalte informiert, es wurde eine schriftliche Einverständniserklärung von Erziehungsberechtigten und den Patienten eingeholt. Alle Aspekte der Studie wurden in Übereinstimmung mit der Deklaration von Helsinki (Version vom Oktober 2013) durchgeführt.

### Patienten

140 Kinder und Jugendliche nahmen von Januar 2018 bis Juni 2022 am interdisziplinären multimodalen ambulanten Therapieprogramm DreKiP (Dresdner Kinder- und Jugendkopfschmerzprogramm) teil. Ein Teil der hier auf geschlechterspezifische Therapieantwort untersuchten Kohorte wurde bereits in der Gesamtheit ausgewertet und publiziert [22]. Die Therapiegruppen wurden nach dem Alter gebildet (9- bis 10-Jährige, 11- bis 13-Jährige, 14- bis 16-Jährige, 17- bis 19-Jährige). Die Daten der Patienten wurden prospektiv erhoben: zu Beginn des Programms (T0), 6 (T1) und 12 (T2) Monate nach dessen Abschluss. Die Analyse basiert auf den Daten von 91 Patienten, die zu T0 und mindestens einem weiteren Zeitpunkt ihre Daten zur Verfügung stellten. Die Therapiegruppen wurden in Zusammenarbeit zwischen der Kopfschmerzambulanz des UniversitätsSchmerzCentrums, der Abteilung für Neuropädiatrie und der Klinik für Kinder- und Jugendmedizin der Universität Dresden organisiert. Die Patienten erhielten vor Programmteilnahme ein standardisiertes Assessment und eine Diagnose nach den Kriterien der International Classification of Headache Disorders (ICHD-III) [24] durch ein interdisziplinäres Team aus den Fachrichtungen Pädiatrie, Neurologie, Schmerzmedizin und Kinder- und Jugendlichenpsychotherapie. Neben ausführlicher Anamnese und klinischer Untersuchung sowie bei Bedarf apparativen Zusatzuntersuchungen wurden Fragebögen zum Persönlichkeitsstil, zum Verhalten, zu Angst- und Depressionssymptomen erfasst (Deutsche Schulalter-Formen der Child Behavior Checklist CBCL nach Achenbach).

Patienten mit der Diagnose einer primären Kopfschmerzerkrankung nach den ICHD-III-Kriterien [24] seit mindestens 6 Monaten im Alter von 9 bis 18 Jahren, mit kopfschmerzbedingten Einschränkungen der Alltagsaktivitäten und des Schulbesuchs sowie ausreichender Therapiemotivation wurden in das Programm aufgenommen.

### Interdisziplinäres multimodales ambulantes Therapieprogramm DreKiP für Kinder und Jugendliche

DreKiP besteht aus 8 Therapiemodulen für Patienten, darunter Kopfschmerzaufklärung, Stressbewältigung, Entspannungstechniken, körperliche Fitness, Klettertherapie als Selbstwirksamkeitstraining, Kunsttherapie, Riechtraining als Edukation zum Einfluss von Sinnesreizen und Achtsamkeitstraining [siehe [22, 23]]. Elternworkshops mit dem Schwerpunkt Edukation finden an 4 Terminen parallel statt.

DreKiP wird über 2–3 Monate an Werktagen nachmittags oder Sonnabenden vormittags durchgeführt. Jede Gruppe besteht aus 4–8 Patienten einer Altersgruppe. Die Patienten erhalten insgesamt 15 Therapiestunden und die Eltern 7 h. Ziel des Programms ist es, die Patienten und ihre Familien über Kopfschmerzen und mögliche Behandlungen aufzuklären, die Verbesserung des Umgangs mit Stress zu erzielen, Entspannungstechniken und Defokussierungsübungen zu erlernen und im Alltag zu implementieren. Die körperliche Aktivierung wird in Form von Teamsport und an der Kletterwand gefördert, die Selbstwirksamkeit wird in der Kletter- aber auch in der Sicherungsposition entwickelt. Detaillierte Therapieinhalte der 8 Module sowie der Aufbaumodule 6 und 12 Monate nach dem Programmende finden sich bei Sobe et al. [22].

### Klinische Daten und Fragebögen

Klinische Daten zu Kopfschmerzdiagnose, Begleiterkrankungen, Kopfschmerzfrequenz, -intensität und Einnahme von Analgetika sowie Fragebögen wurden prospektiv erhoben. Die Kopfschmerzintensität wurde mit einer fünfstufigen Likert-Skala (0 = keine, 1 = geringe, 2 = mittlere, 3 = starke, 4 = stärkste vorstellbare Kopfschmerzintensität) angegeben. Die Analgetikaeinnahme wurde mit 0 = keine Analgetikaeinnahme, 1 = mindestens 1 × pro Monat und 2 = mehr als 1 × pro Woche angegeben. Verwendet wurden der Pediatric Migraine Disability Assessment Score (PedMIDAS, Bereich 0–240 Punkte) [25] und der Pediatric Pain Disability Score (P-PDI) [26]. Der PedMIDAS ist ein validierter Fragebogen zur Quantifizierung von Funktionseinschränkungen aufgrund von Kopfschmerzen über einen Zeitraum von 3 Monaten. Summenwerte > 50 stehen für schwere, 31–50 für mittelschwere, 11–30 für leichte und 0–10 für keine oder geringe Beeinträchtigung aufgrund von Kopfschmerzen. P‑PDI ist ein Fragebogen zur Selbstbeurteilung von schmerzbedingten Beeinträchtigungen bei Kindern und Jugendlichen mit chronischen Schmerzen. Punktwerte von 12–60 werden erreicht, ein hoher Punktwert entspricht großer schmerzbedingter Einschränkung der Alltagsaktivitäten.

### Statistische Verfahren und Datenanalyse

Die Datenanalyse wurde mit SPSS vs. 29 (SPSS Inc., Chicago, IL, USA) durchgeführt. Alle Variablen mit metrischem Skalenniveau wurden auf Normalverteilung getestet, wofür der Shapiro-Wilk-Test angewendet wurde. Kategoriale Variablen wurden als Häufigkeiten und Prozentsätze zusammengefasst; kontinuierliche Variablen wurden als Mittelwerte (MW) ± Standardabweichungen (SD) oder Mediane (mit Interquartilsbereich) angegeben. Um Gruppenunterschiede zu vergleichen, wurden je nach Fall der t‑Test für unabhängige Stichproben oder gepaarte Stichproben, der Mann-Whitney-U-Test (MWU) oder der Wilcoxon-Sign-Rank-Test verwendet.

Bei Hypothesentests wurden *p*-Werte von weniger als 0,05 (zweiseitig) als statistisch signifikant angesehen. Vergleiche über mehrere Zeitpunkte wurde mittels zweifaktorieller Varianzanalyse mit Messwiederholungen (ANOVA) ermittelt, bei multiplem Testen wurde die Bonferroni-Korrektur genutzt.

Der Zusammenhang zwischen Geschlecht und Vorhandensein von Begleiterkrankungen wurde mit dem exakten Test von Fisher untersucht. Die Auswirkung des Alters auf das Vorhandensein von Begleiterkrankungen wurde mittels logistischer Regression untersucht.

## Ergebnisse

### Patientencharakteristika

91 Patienten stellten vollständige Daten zu T0 und mindestens einem Folgezeitpunkt zur Verfügung, bei 49 Patienten gab es keine Folgedaten bzw. nur unvollständige Ausgangsdaten. Gründe dafür waren fehlende Einverständniserklärung, Interessenverlust, Umzug, stationäre Therapie, organisatorische Einschränkungen. Der Vergleich der Ausgangsdaten für Patienten, die ihre Daten im Verlauf zur Verfügung stellten, und mit denen, die dies nicht taten, zeigte im Median eine höhere kopfschmerzbedingte Einschränkung der Alltagsfähigkeit zu Behandlungsbeginn bei 49 Patienten ohne Verlaufsdaten (Patienten, die keine Folgedaten zur Verfügung stellten: PedMIDAS: MW 60,5 Pkt., Median 49,5; IQR [17,25; 99]; Patienten, die in die Auswertung eingeschlossen wurden: PedMIDAS: MW 38, Median 28, IQR [10; 46]) Im Vergleich der Mittelwerte zeigte sich ein signifikanter Unterschied (MWU: 959,500 Z = −1,97 *p* = 0,049).

Wir untersuchten 91 Patienten, davon 65 weiblich (71,4 %) und 26 männlich (28,6 %) im Alter von 9–19 Jahren (Median 15 Jahre, Tab. 1). Für alle Teilnehmer wurde mindestens eine primäre Kopfschmerzerkrankung diagnostiziert (ICHD-III-Kriterien) [24]. 18 Patienten (19,8 %) hatten eine episodische Migräne ohne Aura (eMoA), 9 Patienten (9,9 %) eine episodische Migräne mit Aura (eMmA), 10 Patienten (11 %) eine chronische Migräne (cM), 16 Patienten (17,6 %) zeigten episodische Kopfschmerzen vom Spannungstyp (eST), 8 Patienten (8,8 %) chronische Kopfschmerzen vom Spannungstyp (cST), 9 Patienten (9,9 %) chronische Kopfschmerzen vom Spannungstyp und eine episodische Migräne (cST/eM) und 21 Patienten (23,1 %) zeigten Mischbilder aus episodischer Migräne und episodischen Kopfschmerzen vom Spannungstyp (eM/eST).

**Table 1:** 

	Weiblich (%)	Männlich (%)
*N*	65 (71,4)	26 (28,6)
Alter: < 14 Jahre	26 (61,9)	16 (38)
Alter: ≥ 14 Jahre	39 (80)	10 (20)
Episodische Migräne ohne Aura (eMoA)	10 (55)	8 (45)
Episodische Migräne mit Aura (eMmA)	6 (67)	3 (33)
Chronische Migräne (cM)	8 (80)	2 (20)
Episodischer Spannungskopfschmerz (eST)	12 (75)	4 (25)
Chronischer Spannungskopfschmerz (cST)	6 (75)	2 (25)
Chronischer Spannungskopfschmerz mit episodischer Migräne (cST/eM)	6 (67)	3 (33)
Mischbild episodische Migräne mit episodischem Spannungskopfschmerz (eM/eST)	17 (81)	4 (19)

**Figure 1:**
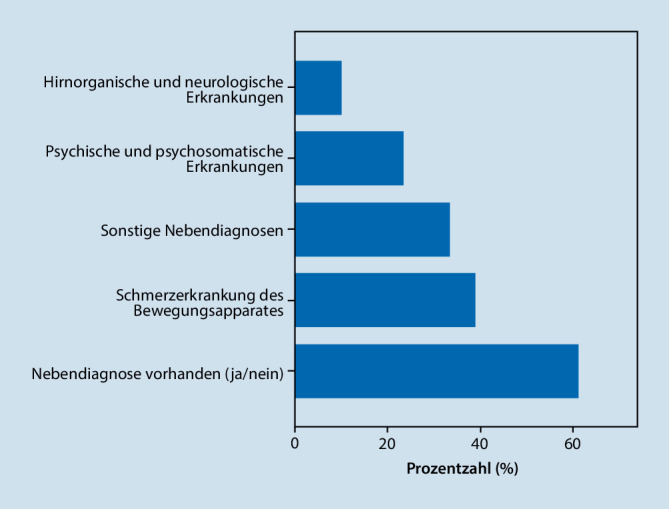

Von den 91 in die Analyse eingegangenen Patienten lag bei 55 (60,4 %) mindestens eine Begleiterkrankung vor (Abb. 1). Am häufigsten traten komorbid Schmerzerkrankungen des Bewegungsapparates (38,5 %) und psychische/psychosomatische Erkrankungen (23,1 %) auf. Zusammengefasst als sonstige Nebendiagnosen wurden unter anderem endokrinologische Erkrankungen, Erkrankungen aus dem atopischen Formenkreis und ophthalmologische Erkrankungen (33 %). Neurologische und hirnorganische Begleiterkrankungen lagen bei 9,9 % der Teilnehmenden vor.

Das Ergebnis der logistischen Regression zeigte einen positiven Zusammenhang zwischen dem Alter der Patienten und dem Vorhandensein einer schmerzhaften Erkrankung des Bewegungsapparats (OR = 1,52, 95 % CI [1,18, 2,07], *p* = 0,003). Ein Zusammenhang zwischen dem Vorhandensein von Schmerzerkrankungen und Geschlecht zeigte sich nicht. Weiterhin wies die logistische Regression einen positiven Zusammenhang zwischen dem Alter und dem Vorhandensein einer psychischen/psychosomatischen Erkrankung aus (OR = 2,11, 95 % CI [1,30, 4,13]), aber keinen Zusammenhang zwischen dem Vorhandensein einer solchen Erkrankung mit dem Geschlecht. Die Verteilung der Kopfschmerzdiagnosen unterschied sich nicht zwischen den Geschlechtern (*χ*^2^ (3) = 2,87, *p* = 0,412).

### Vergleich von Mädchen und Jungen mit primären Kopfschmerzen zu Programmbeginn

Eine Übersicht der Daten für Mädchen und Jungen zu Therapiebeginn zeigt Tab. 2.

**Table 2:** 

	Mädchen (M, [IQR])	Jungen (M, [IQR])	Vergleich
Kopfschmerzfrequenz (in Tagen pro letzte 3 Monate) *n* = 88	M: 42,5 [17,5/90]	M: 20 [11,5/45]	MWU, z = 2,06; *p* = 0,040
Kopfschmerzintensität *n* = 85	M: 2 [2/3]	M: 3 [2/3]	MWU, z = −1,09; *p* = 0,27
Analgetikaeinnahme *n* = 87	M: 1 [1/1]	M: 1 [1/2]	χ^2(2) = 2,85, *p* = 0,241
PedMIDAS *n* = 91	M: 28 [12/50,5]	M: 21 [6,5/41]	MWU, z = 0,954, *p* = 0,343
P‑PDI *n* = 79	M: 34 [27/37]	M: 31,5 [22,5/37]	MWU, z = −0,92; *p* = 0,35

*PedMIDAS* Pediatric Migraine Disability Score, *P‑PDI* Pediatric Pain Disability Index, *M* Median, *[IQR]* interquartile range, *MWU* Mann-Whitney-U-Test

Bei 88 der untersuchten Patienten lagen uns Daten zur Kopfschmerzfrequenz zu Programmbeginn vor. Mädchen hatten im Median 42,5 Kopfschmerztage in den letzten 3 Monaten, Jungen im Median 20. Damit lag bei Mädchen eine höhere Kopfschmerzfrequenz als bei Jungen vor (MWU, z = 2,06; *p* = 0,040).

Zur Kopfschmerzintensität lagen uns zu Programmbeginn bei 85 Patienten die Daten vor. Mädchen zeigten im Median einen Punktwert von 2, Jungen von 3. Im Vergleich zwischen den Geschlechtern zeigte sich kein signifikanter Unterschied (MWU, z = −1,09; *p* = 0,27).

Ein statistisch signifikanter Geschlechterunterschied in der Analgetikaeinnahme konnte zu Programmbeginn nicht nachgewiesen werden (T0: *χ*^2^(2) = 2,85; *p* = 0,241).

Im Mittel lag der PedMIDAS-Score bei 38 Punkten (SD = 38,3; Median = 28, IQR[10;49]). Diese Daten lagen zu Beginn des Programmes für 91 Mädchen und Jungen vor. Mädchen zeigten numerisch eine etwas höhere kopfschmerzbedingte Beeinträchtigung als Jungen (PedMIDAS Mediane, T0: ♀ 28, ♂ 21). Allerdings war der Unterschied zu Programmbeginn nicht signifikant (Mann-Whitney-U-Test (MWU): z = 0,954; *p* = 0,343).

Die mittlere schmerzbedingte Einschränkung der Alltagsfähigkeit im P‑PDI-Score für Mädchen und Jungen zusammen lag zu Programmbeginn bei 33 Punkten (SD = 8,51; Median = 34, IQR [26;37]). Dazu waren zu Therapiebeginn die Scores von 79 Patienten vorhanden. Im Geschlechtervergleich zeigten sich höhere P‑PDI-Werte bei Mädchen (P-PDI Mediane T0: ♀ 34, ♂ 31,5). Dieser Unterschied war statistisch nicht signifikant (Tab. 2).

### Therapieeffekte im Geschlechtervergleich

#### Kopfschmerzfrequenz

Zum Verlauf der Kopfschmerzfrequenz nach der Therapie konnte eine Varianzanalyse für 46 Patienten, für welche zu allen 3 Messzeitpunkten Daten vorlagen, gerechnet werden (Abb. S1 im Online-Zusatzmaterial). Ein signifikanter Effekt der Zeit auf die Kopfschmerzfrequenz konnte nachgewiesen werden. Ein signifikanter Effekt des Geschlechts fand sich nicht. Ebenso war keine signifikante Interaktion zwischen Zeit und Geschlecht darstellbar (F (2;88) = 0,869; *p* = 0,423; Tab. 3). Dabei zeigte der Bonferroni-korrigierte Post-hoc-Test eine signifikante Reduktion zwischen T0 und T1 (*p* = 0,016; M_Diff_ = 10,24; 95 % CI [1,5;19,0]), sowie zwischen T0 und T2 (*p* < 0,0001; M_Diff_ = 17,32; 95 % CI [8,6;26,1]). Nach dem Programm, zum Zeitpunkt T1 und T2 nahm die Differenz der Kopfschmerzfrequenz zwischen Mädchen und Jungen zu, damit zeigten Jungen im Mittel, jedoch ohne statistische Signifikanz zu erreichen, eine größere Kopfschmerzreduktion als Mädchen.

**Table 3:** 

	Haupteffekt der Zeit	Haupteffekt des Geschlechts	Interaktionseffekt Zeit & Geschlecht
*Kopfschmerzfrequenz*	Ja	Nein	Nein
(*n* = 46)	F (2;88) =11,840; *p* < 0,001	F (1;44) =1,041; *p* = 0,313	F (2;88) =0,869; *p* = 0,423
*Kopfschmerzintensitä*t	Nein	Nein	Nein
(*n* = 35)	F (2;66) =2,166; *p* = 0,123	F (1;33) =1,668; *p* = 0,206	F (2;66) =1,258; *p* = 0,291
*PedMidas*	Ja	Nein	Nein
(*n* = 48)	F (2;92) =5,451; *p* = 0,006	F (1;46) =3,526; *p* = 0,067	F (2;92) =0,194; *p* = 0,824
*P‑PDI*	Nein	Nein	Nein
(*n* = 36)	F (2,68) = 1,251; *p* = 0,293	F (1,34) = 3,386; *p* = 0,074	F (2, 68) = 2,125; *p* = 0,127

*PedMIDAS* Pediatric Migraine Disability Score, *P‑PDI* Pediatric Pain Disability Index

**Figure 2:**
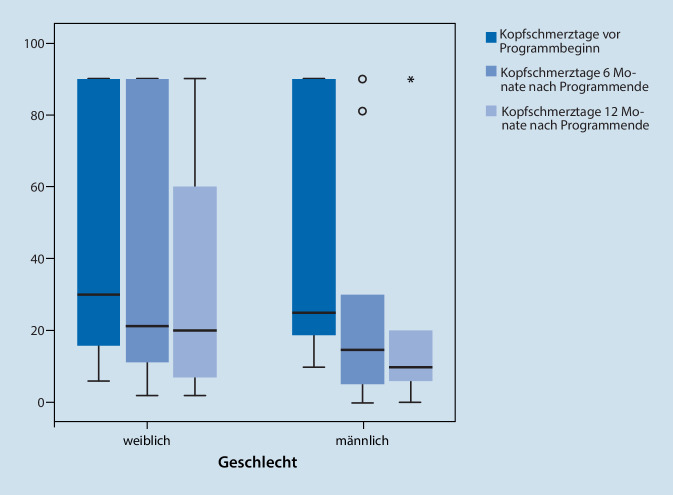

Bei Auswertung aller verfügbaren Verlaufsdaten (T1: *n* = 77, T2: *n* = 58) zeigten Mädchen zu allen Zeitpunkten eine höhere Kopfschmerzfrequenz als Jungen (T1: MWU: z = 2,54; *p* = 0,011 T2: MWU, z = 2,12; *p* = 0,035). Sowohl für Mädchen als auch für Jungen war die Kopfschmerzfrequenz zu T1 und T2 reduziert (Kopfschmerztage der vergangenen 3 Monate, Mädchen Median T0:42,5 Tage; T1: 31,5 Tage; T2: 28 Tage; Jungen Median: T0: 20 Tage; T1: 12 Tage; T2: 9 Tage, Abb. 2).

#### Kopfschmerzintensität

Zum Verlauf der Kopfschmerzintensität konnte eine Varianzanalyse für nur 35 Patienten mit Daten zu allen Messzeitpunkten gerechnet werden. In der Varianzanalyse konnten keine signifikanten Haupt- und Interaktionseffekte nachgewiesen werden (F (2;66) = 2,649; *p* = 0,078) (Tab. 3, Abb. S2 im Online-Zusatzmaterial).

**Figure 3:**
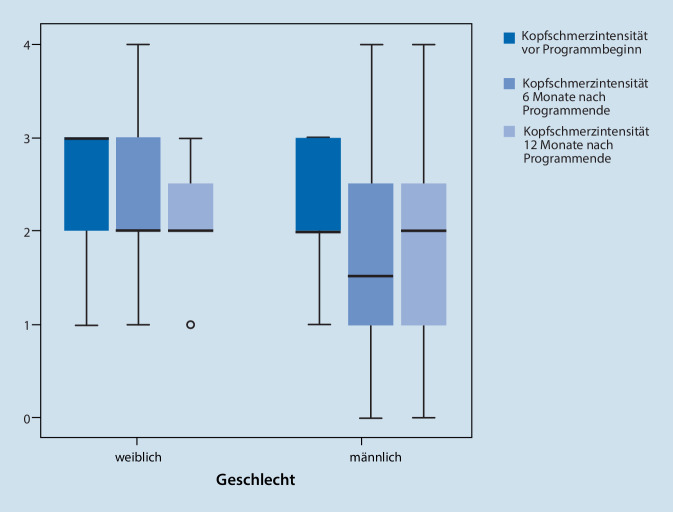

Bei Auswertung aller verfügbaren Verlaufsdaten (T0: *n* = 85, T1: *n* = 69, T2: *n* = 47) zeigt sich numerisch eine Reduktion der Kopfschmerzintensität (Abb. 3).

#### Analgetikaeinnahme

Nach dem Therapieprogramm zeigten die Patienten (Jungen und Mädchen zusammen) eine Reduktion der Analgetikaeinnahme. Dies lässt sich aus folgenden Odds-Ratios (nach Bonferroni-Korrektur) ableiten: Vergleich zwischen Zeitpunkt T1 und T0: OR = 0,22, 95 % CI [0,02; 1,97], *p* = 0,293; zwischen Zeitpunkt T2 und T0: OR = 0,10, 95 % CI [0,01; 0,94], *p* = 0,042; Zeitpunkt T2 im Vergleich zu T1: OR = 0,45, 95 % CI [0,07; 2,78]; *p* = 0,883.

**Figure 4:**
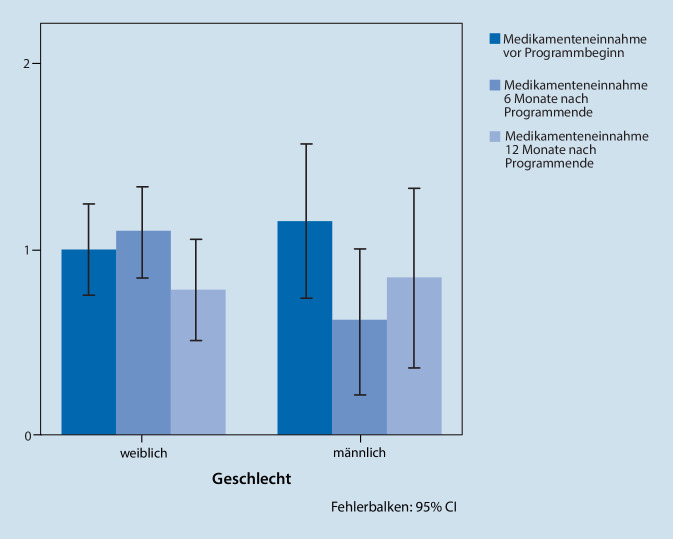

Die Daten weisen auch Geschlechterunterschiede bei der Analgetikaeinnahme aus (Abb. 4). Basierend auf den Kategorien zur Einnahmefrequenz der Analgetika (0 = keine Analgetikaeinnahme, 1 = mindestens 1 × pro Monat und 2 = mehr als 1 × pro Woche) lag zum Zeitpunkt T1 ein Unterschied bei der Analgetikaeinnahme vor, Mädchen nahmen signifikant mehr Analgetika als Jungen ein (T1: *χ*^2^(2) = 7,69; *p* = 0,021; T2: *χ*^2^(2) = 0,21; *p* = 0,902).

Zur weiteren Untersuchung der Geschlechterunterschiede wurde eine logistische Regression mit gemischten Effekten genutzt. Dafür erhoben wir, wie viele Mädchen und Jungen zu den verschiedenen Untersuchungszeitpunkten Analgetika einnahmen bzw. nicht einnahmen (Antwortvariable 0 = keine Analgetikaeinnahme, 1 = Analgetikaeinnahme). Hier zeigte sich, dass zu allen Zeitpunkten mehr Mädchen Analgetika einnahmen als Jungen (OR = 1,72, 95 % CI [0,11; 26,20]; *p* = 0,698). Sowohl Mädchen, als auch Jungen reduzierten im Verlauf der Zeit die Analgetikaeinnahme.

#### Kopfschmerzbedingte Einschränkung der Alltagsaktivität (PedMIDAS)

**Figure 5:**
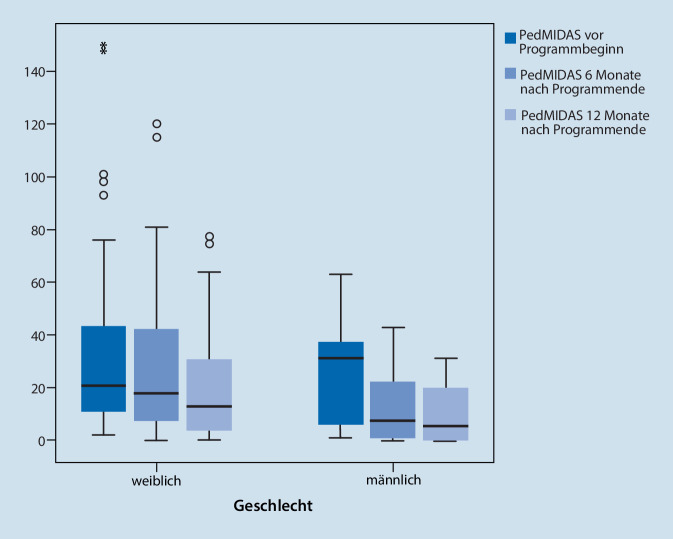

Eine Varianzanalyse wurde für 48 Patienten durchgeführt. Dabei zeigte sich ein signifikanter Haupteffekt von Zeit im PedMIDAS-Summenwert (F (2;92) = 5,340; *p* = 0,006), jedoch kein Haupteffekt des Geschlechts und auch kein Interaktionseffekt von Zeit und Geschlecht (Tab. 3, Abb. S3 im Online-Zusatzmaterial). Der Bonferroni-korrigierte Post-hoc-Test zeigte keine signifikante Reduktion zwischen T0 und T1 (M_Diff_ = −8,69; 95 % CI [−19,9;2,53]; *p* = 0,186), aber eine signifikante Reduktion zwischen T0 und T2 (M_Diff_ = −15,13; 95 % CI [−26,3;−3,91]; *p* = 0,004). Bei Auswertung aller verfügbaren Verlaufsdaten (T0: *n* = 91, T1: *n* = 77, T2: *n* = 58, Abb. 5) zeigten Mädchen zu allen Zeitpunkten eine höhere kopfschmerzbedingte Beeinträchtigung als Jungen, allerdings war der Unterschied zwischen T0 und T1 nicht signifikant (T0, MWU: z = 0,954; *p* = 0,343; T1, MWU: z = 1,83; *p* = 0,069). Lediglich 12 Monate nach Therapieende erreichte der Unterschied der kopfschmerzbedingten Alltagseinschränkung auch statistische Signifikanz (T2, MWU: z = 2,31; *p* = 0,021).

#### Schmerzbedingte Einschränkung der Alltagsaktivität (P-PDI)

Eine Varianzanalyse wurde für 36 Patienten mit vollständig vorhandenen Daten zu T0, T1 und T2 durchgeführt. Hier zeigte sich lediglich ein Haupteffekt von Zeit (F (2;68) = 5,229; *p* = 0,008), kein Haupteffekt des Geschlechts und kein signifikanter Interaktionseffekt zwischen Zeit und Geschlecht (F (2; 68) = 2,125; *p* = 0,127; Tab. 3, Abb. S4 im Online-Zusatzmaterial).

In der Patientengruppe zeigte sich eine signifikante Reduktion im -PPDI-Score nach 6 Monaten (Wilcoxon-Test, z = −4,04; *p* < 0,001), und nach 12 Monaten (Wilcoxon-Test, z = −3,87; *p* < 0,001), jeweils im Vergleich zum Wert vor Programmbeginn.

**Figure 6:**
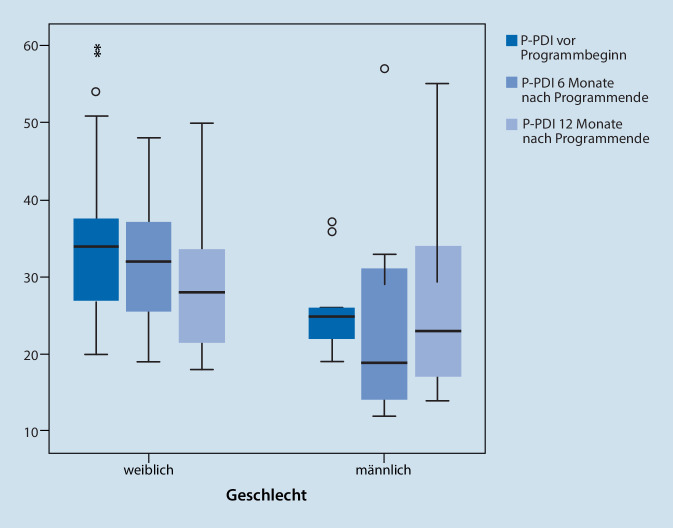

Im Geschlechtervergleich unter Einbeziehung aller verfügbarer Daten (T0: *n* = 79, T1: *n* = 71, T2: *n* = 55) zeigten sich zu allen Messzeitpunkten numerisch höhere P‑PDI-Werte von Mädchen. Jungen waren demnach weniger durch Schmerzen in ihrer Alltagsfunktion beeinträchtigt als Mädchen (Abb. 6).

## Diskussion

Effekte interdisziplinärer multimodaler Therapieansätze für primäre Kopfschmerzerkrankungen im Kindes- und Jugendalter wurden in verschiedenen Studien nachgewiesen [22, 27, 28]. Erste Auswertungen zeigten auch positive Ergebnisse des ambulanten Therapieprogramms DreKiP für die Zielgruppe Kinder und Jugendliche mit primären Kopfschmerzen [22].

In der aktuellen Datenanalyse wurde die Kohorte auf geschlechterspezifische Therapieeffekte untersucht. Die aus epidemiologischen Untersuchungen bekannte erhöhte Prävalenz für Migräne und Kopfschmerz vom Spannungstyp bei Mädchen und Frauen ab der Pubertät bestätigt sich auch in unserer Patientenkohorte [29]. Teilnehmende Patienten waren in 71,4 % Mädchen. Weiterhin waren Patienten der Altersgruppen ≥ 14 Jahre häufiger im Programm vertreten als Patienten < 14 Jahre. Das korrespondiert zur erhöhten Prävalenz von Kopfschmerzen ab dem Jugendalter [7, 13, 30, 31].

Für beide Geschlechter zeigt die aktuelle Analyse Therapieeffekte des Gruppenprogramms. Eine Reduktion der Kopfschmerzfrequenz und der kopfschmerzbedingten Alltagseinschränkung wurde sowohl für Mädchen als auch für Jungen mit primären Kopfschmerzen erreicht. Jedoch fallen Mädchen zu allen Untersuchungszeitpunkten durch höhere Kopfschmerzfrequenz und größere kopfschmerzbedingte Einschränkung der Alltagsfähigkeit im Vergleich zu Jungen auf, wenngleich die Unterschiede nicht immer Signifikanz erreichten.

Diese Ergebnisse passen gut zu verschiedenen veröffentlichten Daten internationaler Kopfschmerzstudien an Erwachsenen. So weisen aktuelle Daten von Verhagen et al., die mittels elektronischer Kopfschmerztagebücher erhoben wurden, bei Erwachsenen einen Geschlechterunterschied der Migräneausprägung auf. Dabei zeigte sich bei 1347 Frauen und 284 Männern mit Migräne, dass die Attacken bei Frauen länger dauern und mit mehr Begleitsymptomen wie Phono‑, Photophobie und Übelkeit einhergingen [32]. Eine große amerikanische Studie mit über 162.000 Teilnehmern (Alter ≥ 12 Jahre) zeigte bei weiblichen Teilnehmenden mit Migräne eine stärkere Beeinträchtigung und häufigere Inanspruchnahme des Gesundheitssystems im Vergleich zu männlichen Teilnehmenden [33]. Daten einer israelischen Spezialambulanz für Kinderkopfschmerz zeigten für Mädchen höhere Kopfschmerzfrequenzen und häufigeres Auftreten von chronischer Migräne [34]. Ähnliche Ergebnisse weist unserer Patientenkohorte auf. Darüber hinaus gibt es eine Geschlechterdifferenz in der Einnahme von Analgetika, wobei Mädchen häufiger Analgetika nutzen. Möglicherweise geschieht das aufgrund höherer Kopfschmerzfrequenz und größerer kopfschmerzbedingter Einschränkung, jedoch wurde diese Kausalität hier nicht getestet.

Die Prävalenz von Kopfschmerzen bei Mädchen und Frauen ab der Pubertät ist etwa drei- bis viermal so hoch im Vergleich zu Männern [18, 42, 43]. Die Ursachen sind bis heute nicht umfassend geklärt und annehmbar multifaktoriell. Als ein wichtiger Faktor wurde die zyklische Änderung des Östrogenserumspiegels bei Frauen identifiziert. So wurden Östrogene als Regulator verschiedener – auch pronozizeptiver – Transmitter erkannt, u. a. Serotonin und Glutamat. Im Tiermodell konnten auch direkte Effekte von Östrogen auf die Modulation der Nozizeption via Transient-Receptor-Potential-Vanilloid 1(TRPV1)-Rezeptoren im trigeminalen System gezeigt werden. Studien deuten darauf hin, dass weibliche Geschlechtshormone die Prozessierung nozizeptiver Reize im zentralen Nervensystem bei Migräne beeinflussen [44, 45]. Eine Studie an 39 gesunden Probandinnen mittels quantitativ sensorischer Testung zeigte eine zyklusabhängige Varianz der sensorischen und Schmerzwahrnehmungsschwellen, sowohl im trigeminalen Areal (V2) als auch extratrigeminal (Unterarm) [47]. Für extratrigeminale Körperregionen existieren umfangreichen Untersuchungen, die menstruell erniedrigte mechanische Schmerzschwellen nachweisen [48–50]. Darüber hinaus wurde für viele schmerzhafte Erkrankungen wie z. B. temporomandibuläre Schmerzen oder Migräne eine hormon-/zyklusabhängige Schwankung der klinischen Präsentation beschrieben [51, 52]. Passend dazu konnte in einer Studie an 40 gesunden Frauen gezeigt werden, dass individuell höhere Testosteronspiegel mit einer höheren Toleranz gegen mechanische Schmerzreize einhergingen [53].

Zusätzlich wurden weitere Faktoren wie genetische Polymorphismen, strukturelle Unterschiede z. B. der Inselrinde [54], funktionell verschiedene Aktivitäten des Salienznetzwerks und psychosoziale Stressoren aufgezeigt [55].

Zu den sozialen und umweltbedingten Faktoren, die negativ auf den Verlauf von Migräne einwirken können, zählen Gewalt und negative Erfahrungen in der Entwicklung, wie z. B. soziale Überforderung [56]. Da überwiegend Mädchen und Frauen Opfer dieser Arten von Missbrauch sind, erscheint die Bedeutung des Geschlechts für die Ausbildung wiederkehrender Kopfschmerzen und Migräne noch deutlicher.

In einer Studie zu geschlechterspezifischen psychosozialen Einflüssen von Kopfschmerzen bei Kindern und Jugendlichen zeigten sich bei den Mädchen Schulstress und dysfunktionale Stress-Coping-Strategien als prädisponierende Faktoren für Kopfschmerz im Vergleich zu Jungen [46, 57].

Insgesamt nehmen Kinder und Jugendliche mit höherem Alter häufiger am Therapieprogramm teil. Diese Beobachtung kann mit Daten nationaler, aber auch internationaler Studien übereinstimmen, welche eine höhere Kopfschmerzfrequenz mit steigendem Alter der Kinder und Jugendlichen nachweisen [7, 29, 35–37]. Hier wurde ein Peak der Frequenz für Migräne und Kopfschmerz vom Spannungstyp ab dem 15. Lebensjahr bis zum 50. Lebensjahr gezeigt [29, 35]. Auch verschiedene Studien in Populationen von Kindern und Jugendlichen weisen auf die altersabhängige Prävalenz von Kopfschmerzen hin [38, 39].

In unserer Untersuchung zeigen wir, dass Mädchen zwar schwerer von Kopfschmerzen betroffen sind, jedoch ebenso gut auf das Therapieprogramm ansprechen wie Jungen. Hier besteht ein Unterschied zu Therapieeffekten bei erwachsenen Populationen. Beispielsweise wurde in der Therapie der chronischen Migräne mit Onabotulinumtoxin A gezeigt, dass die Kopfschmerzreduktion bei Frauen im Vergleich zu Männern weniger deutlich ausfällt [59].

Für Kinder und Jugendliche mit primären Kopfschmerzen resultierte unser Therapieprogramm in einer deutlichen Kopfschmerzreduktion. Dies kann auf unmittelbaren Effekten von Edukation und verbesserter Akuttherapie beruhen. Auch ist altersbedingt eine geringere Erkrankungsdauer zu erwarten und damit eine geringere Chronifizierung. Beispielsweise weisen Daten eine Dauer der Migräneerkrankung von > 30 Jahren als negativen prädiktiven Faktor für das Ansprechen auf eine Therapie mit Onabotulinumtoxin A aus [60]. Neurophysiologische Prozesse, die die Basis für kortikale Plastizität bilden, sind typischerweise im Kindes- und Jugendalter am aktivsten [61]. Dies kann ein weiterer Grund für schnelles Therapieansprechen bei Kindern und Jugendlichen sein. Möglicherweise beruht es auch auf funktionellen Unterschieden zentralnervöser Netzwerke, wie sie für Kinder und Jugendliche im Vergleich zu jungen Erwachsenen mit Migräne gezeigt wurden [40].

Interessanterweise gibt es in verschiedenen klinischen Studien zur Migräneprophylaxe bei Kindern und Jugendlichen ähnliche Entwicklungen der Kopfschmerzhäufigkeit. Dabei zeigt sich eine relativ zügige Reduktion der Kopfschmerzfrequenz, unabhängig von der Intervention [58]. Bisher sind die Mechanismen dieser klinisch relevanten Verbesserung noch nicht ausreichend untersucht. Es ist möglich, dass neben Placebophänomenen andere physiologische Effekte eine Rolle spielen [41].

Der Einfluss der Kopfschmerzdiagnosen auf die Reduktion der Kopfschmerztage nach dem Therapieprogramm ist statistisch nicht signifikant. Allerdings haben fast 74 % der Patienten eine Migränediagnose, als alleinigen primären Kopfschmerz oder in Kombination mit einem Kopfschmerz vom Spannungstyp. Demzufolge ist eine Auswertung in der Aussage eingeschränkt. Deutlich wird dabei, dass Kinder und Jugendliche mit Migräne einen hohen Leidensdruck haben, der sie nicht nur zum Arzt führt, sondern auch in der Freizeit ein Kopfschmerztherapieprogramm wahrnehmen lässt. Zusammenfassend zeigt sich im Geschlechterunterschied eine stärkere Ausprägung der Kopfschmerzbelastung bei Mädchen, ein Ansprechen auf das multimodale Therapieprogramm DreKiP ist für beide Geschlechter vorhanden.

Die Studie weist als Limitation vor allem fehlende oder unvollständige Follow-up-Daten der teilnehmenden Patienten auf. In diesem Zusammenhang entsteht ein Selektionsbias, da schwer betroffene Patienten seltener Follow-up-Daten zur Verfügung stellen. Aufgrund der unvollständigen Daten wurden mit den vorhandenen Daten unterschiedliche Analysen durchgeführt. Da nur ein Teil der Stichprobe zu allen drei Untersuchungszeitpunkten Daten bereitstellte und somit die Stichprobe für die Varianzanalysen reduziert war, resultierten unterschiedliche, teilweise auch abweichende Ergebnisse der Varianzanalysen im Vergleich zu den paarweise durchgeführten Vergleichen aller pro Untersuchungszeitpunkt vorhanden Daten.

Eine elektronische Erfassung von Patientendaten, z. B. über eine Smartphone-Applikation könnte einen Datenverlust vermindern. Ebenfalls könnten so weitere Fragebögen mit einbezogen werden, z. B. zur Erfassung der Schlafqualität oder von Symptomen für Ängstlichkeit und Depression. Auch konnten in der vorliegenden Untersuchung keine Daten einer Kontroll- oder Wartegruppe hinzugezogen werden. Zur Überprüfung der Ergebnisse ist eine randomisierte und kontrollierte Studie notwendig.

### Supplementary Information





## Abkürzungen

BMI: Body Mass Index
CBCL: Child Behavior Checklist
CGRP: Calcitonin Gene-related Peptide
CI: Konfidenzintervall
cM: chronische Migräne
cST: chronische Kopfschmerzen vom Spanungstyp
eM: episodische Migräne
eMmA: episodische Migräne mit Aura
eMoA: episodische Migräne ohne Aura
eST: episodische Kopfschmerzen vom Spannungstyp
fMRI: functional Magnetic Resonance Imaging
HIT‑6: Headache Impact Test
ICHD‑3: International Classification of Headache Disorders
IQR: Interquartilsrange
MIDAS: Migraine Disability Assessment Score
MW: Mittelwert
MWU: Mann-Whitney-U-Test
OR: Odds-Ratio
PedMIDAS: Pediatric Migraine Disability Assessment Score
P‑PDI: Pediatric Pain Disability Index
SD: Standardabweichung
SPSS: Statistical Package for the Social Sciences
TRPV1: Transient-Receptor-Potential-Vanilloid 1
