# Supplementary material for: Gender-specific results of the Dresden children and adolescents headache program DreKiP
Source: Schmerz. 2023 Sep 22;38(2):107–17. [Article in German] doi: 10.1007/s00482-023-00756-z (PMC10959813; doi:10.1007/s00482-023-00756-z)
Supplement: Supplementary file 1 [file 482_2023_756_MOESM1_ESM.pdf]

Online-Zusatzmaterial

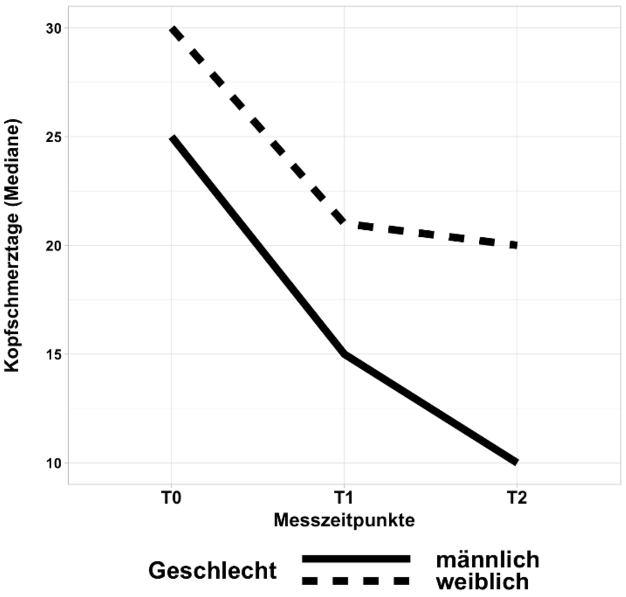

Abb. S1 Kopfschmerzfrequenz im Therapieverlauf für Patienten mit vollständigen Daten zu allen Untersuchungszeitpunkten (n=46; Kopfschmerztage in den letzten 3 Monaten)

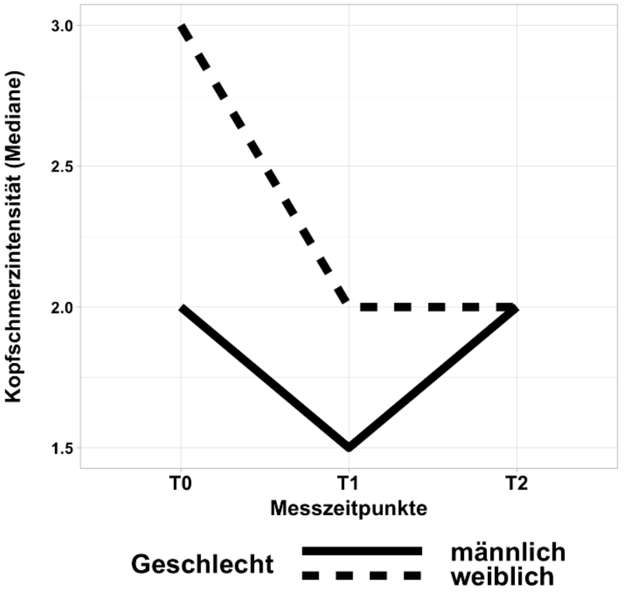

Abb. S2 Kopfschmerzintensität im Therapieverlauf für Patienten mit vollständigen Daten zu allen Untersuchungszeitpunkten (n=35; 0=keine, 1=geringe, 2=mittlere, 3=starke, 4=stärkste vorstellbare Kopfschmerzintensität)

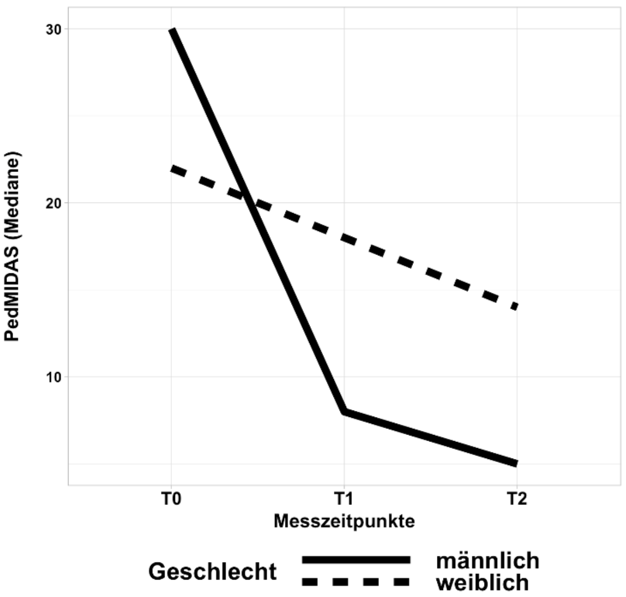

Abb. S3 PedMIDAS im Therapieverlauf für Patienten mit vollständigen Daten zu allen Untersuchungszeitpunkten (PedMIDAS=Pediatric Migraine Disability Score, n=48; Summenwerte >50=schwere, 31-50=mittelschwere, 11-30=leichte, 0-10=keine oder geringe Beeinträchtigung aufgrund von Kopfschmerzen)

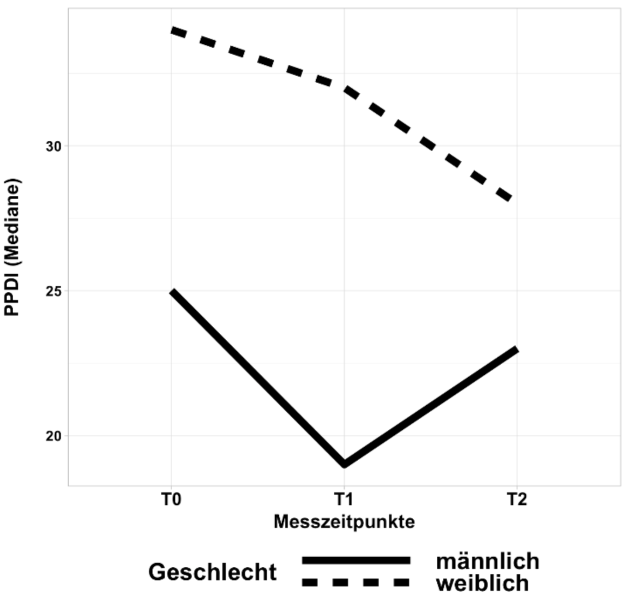

Abb. S4 PPDI im Therapieverlauf für Patienten mit vollständigen Daten zu allen Untersuchungszeitpunkten (PPDI=Pediatric Pain Disability Index, n=36; Summenwerte von 12-60: hoher Punktwert entspricht großer schmerzbedingter Einschränkung der Alltagsaktivitäten)
